# Allergy screening with extract‐based skin prick tests demonstrates higher sensitivity over in vitro molecular allergy testing

**DOI:** 10.1002/clt2.12220

**Published:** 2023-02-05

**Authors:** Tobias Gureczny, Benjamin Heindl, Livia Klug, Felix Wantke, Wolfgang Hemmer, Stefan Wöhrl

**Affiliations:** ^1^ Floridsdorf Allergy Center (FAZ) Vienna Wien Austria; ^2^ Medical University of Vienna Vienna Wien Austria

**Keywords:** allergen component, in vitro, molecular allergology, sensitivity, skin prick test

## Abstract

**Background:**

As extract‐based skin testing as well as in vitro tests for major allergens have their own advantages, both procedures are usually performed in routine settings. In times of shortages in medical staff and supplies, we asked ourselves, how many patients would be underdiagnosed, if only one test could be used.

**Methods:**

In a retrospective analysis, we investigated a cohort of 2646 patients seen by a single physician in a large Austrian outpatient allergy clinic in 2018. Only patients with an allergen source‐specific history and pairs of extract‐based skin prick (SPT) and in vitro molecular allergy tests to major allergens were included.

**Results:**

For all tested allergen sources, sensitivity was higher for SPT than for sIgE‐based molecular allergy testing. Concerning 1006 birch pollen‐allergic patients, 791 (78.6%) had positive results with both tests, while 153 (15.2%) only with the SPT and 62 (6.2%) only with the sIgE to Bet v1. The other allergen sources showed similar results: For house dust mite 816/1120 (72.9%), grass pollen 1077/1416 (76.1%) and cat 433/622 (69.6%) remained test‐positive with both procedures, whereas in 276 (24.6%), 224 (15.8%) and 173 (27.8%) times only the SPT and 28 (2.5%), 115 (8.1%) and 16 (2.6%) times only the sIgE to Der p1/2/23, Phl p1/5 and Fel d1 showed a positive result. Each comparison was statistically significant (each *p* < 0.0001, Chi‐squared test).

**Conclusions:**

Screening for allergy with major molecular allergens has lower sensitivity when compared with extract‐based skin tests. A combination of both is required for an optimal sensitivity.

## 1 INTRODUCTION

In allergology, allergen skin and in vitro tests are widely used for screening purposes. Hence, reliability that is, sensitivity and specificity are important to reliably confirm or rule out IgE‐mediated allergy in large patients' cohorts. In the past, there were studies suggesting a better sensitivity of extract‐based skin prick tests (SPTs) than extract‐based in vitro tests.^1^

Skin testing has always been a standard procedure in allergy diagnosis.^2^ It offers the advantage of giving immediate results that can be discussed with patients without having to wait for laboratory results and without requiring a second appointment for discussing the clinical relevance of the allergy tests. Also, the results of skin tests are quite instructive to patients especially concerning emotional allergens such as pet animals. However, skin testing is an expensive procedure requiring extra‐work force by skilled personal. *Urticaria factitia* (dermatographism) and clinically irrelevant sensitization to pan‐allergens (e.g., profilin) may hamper the reading in a small number of patients. Also, some drugs such as antihistamines, mirtazapine and quetiapine may be the cause for false negative results.^3^ The withdrawal of commercially available SPT reagents has also been a problem reducing availability of important skin test reagents, for example, latex extract.^4^ However, also in vitro tests have been voluntarily withdrawn from the market by manufacturers and supply problems have affected also important molecular allergens for example, the major latex allergens Hev b5 and Hev b6. High total IgE levels might lead to false positive and very low total IgE levels to false negative in vitro tests.^5^

Austria is a fortunate high‐income country, where double allergy testing is available and reimbursed by public healthcare. In this study, we asked ourselves, how many allergy patients would be falsely labeled as NOT suffering from allergy if only one of the methods would be available: (a) extract‐based SPT or (b) molecular allergy based on specific IgE (sIgE) with the major allergens of the most important inhalant allergen sources. We compared pairs of SPT and sIgE to house dust mite, cat, birch and grass pollen concerning sensitivity in patients with a clear‐cut history of clinically relevant allergy to any of the aforementioned allergen sources.

## 2 METHODS

### 2.1 Patients & ethics committee vote

**FIGURE 1:**
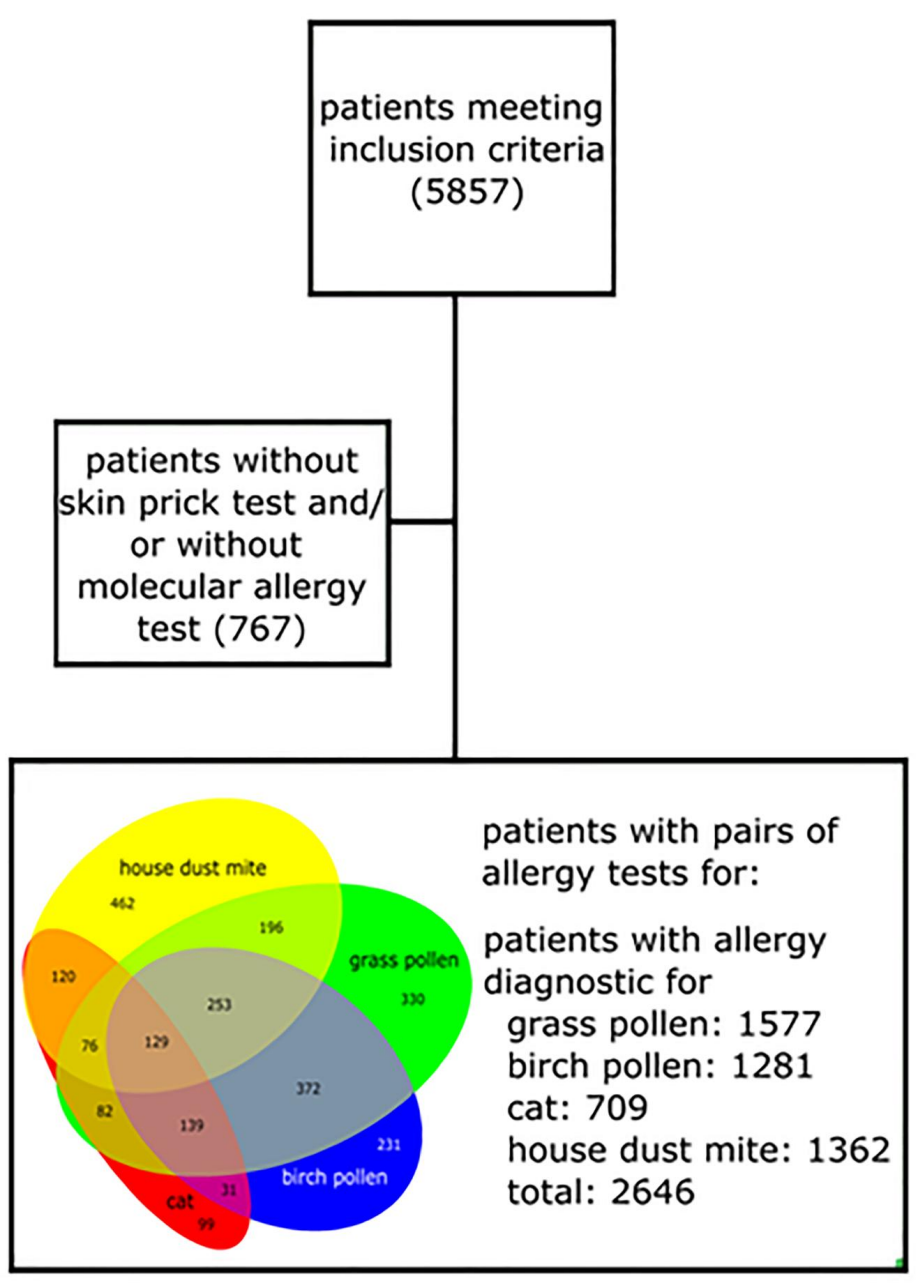
Flow diagram demonstrating the patients meeting all inclusion criteria in a Venn diagram.

A cohort of patients presenting themselves at the Floridsdorf Allergy Center (FAZ) between January 2018 and December 2018 with a history compatible with an IgE‐mediated type 1 allergy to inhalant allergens was included into this study. All patients were seen by a single physician to avoid inter‐observer variation. Only patients with complete pairs of extract‐based SPT and sIgE to the major allergens of cat (Fel d1), birch pollen (Bet v1), grass pollen (Phl p1 & 5), and house dust mite (Der p1/2/23) were included into this study. We chose cat and birch pollen as representants for allergen sources with dominating single major allergens (Fel d1 and Bet v1 respectively) and grass pollen and house dust mite (*Dermatophagoides pteronyssinus*) as representants for complex allergens with more than two dominating major allergens. To exclude double entries of the same patients, only the first visit was considered in patients with several presentations in the year 2018. Of the 5857 available data sets, 2646 patients were finally included into the study (Figure 1).

The patients' data had been retrospectively retrieved from routine patients records and entered into a spreadsheet by TG/BH/LK as part of their medical diploma thesis at the Medical University of Vienna (Ethics committee vote #2213/2018). The informed consent form is provided in the online repository (available in German as S1). The details were already described elsewhere.^6^

### 2.2 Diagnostics

The diagnostic procedure started by obtaining a careful allergen‐specific history by an experienced allergologist. Allergen‐specific details were recorded and allocated to the referring allergen (e.g., cat‐specific symptoms and cat‐ownership, allergic‐rhinoconjunctivitis in March–April [birch pollen], May–July [grass pollen], or every morning [house dust mite]). The SPTs were performed with standard allergen extracts acquired from ALK–Abelló or Bencard/AllergyTherapeutics depending on availability. The SPTs were done with standard SPT‐lancets acquired from the suppliers. Histamine‐dihydrochloride and 0.9% w/v NaCl acted as positive and negative controls, respectively. The wheal sizes were read after 20 min and classified at the time of the reading according to the recommendations of the German guideline^7^: the wheal‐size was categorized into five groups, scaled by their diameter (<3 mm; 3–4 mm; 4–5 mm; 5–6 mm; >6 mm). Molecular allergy tests were performed in all patients with an allergen‐specific history independent of their SPT results. For in vitro tests, we used the industry quasi‐standard ImmunoCAP^®^ 250 from ThermoFisherScientific/Phadia.

### 2.3 Statistical analysis

The data analysis was performed with SPSS (Version 23, IBM). A Chi‐Squared‐Test was used to compare the results of SPT and molecular testing. Wheals with diameters of 3 mm or greater and antibody levels of 0.35 kU/l or higher were considered positive. The ellipsoid Venn diagram for Figure 1 was created with eulerr (https://cran.r‐project.org/web/packages/eulerr/index.html, Version 6.1.1). Other Venn diagrams were created with venn.js (https://github.com/upsetjs/venn.js, Version 1.4.2) for a visual comparison of the results. All graphics were revised later on with Inkscape (https://inkscape.org/de/, Version 1.1). A Spearman's coefficient of rank correlation was used to measure the correlation between wheal size and antibody levels. The significance level for all tests was set at *p* < 0.05.

## 3 RESULTS

### 3.1 Patients

There were 2646 patients, who met the inclusion criteria (Table 1). 53.7% were female and the arithmetic mean age was 32.7 years (*σ* = 18.0 years). Thirty‐five percent of them suffered from allergic rhinoconjunctivitis, 11% from bronchial asthma or allergic cough and 7% from atopic dermatitis. We compared the results of the SPT with the molecular allergy diagnostics from 1281 patients regarding birch pollen allergy, from 1362 patients regarding dust mite allergy, from 1577 patients regarding grass pollen allergy and from 709 patients regarding cat allergy. Some patients suffered from sensitizations to more than one allergen (details in Figure 1). In total, the tests of 2646 patients were compared this way.

**TABLE 1:** Study population

		Female	Male	Total
Patients [*n*, (%)]		1421 (53.7)	1225 (46.2)	2646 (100.0)
Age [x̄+‐σ]		34.72 ± 17.61	30.26 ± 18.22	32.65 ± 18.03
Tested with	Birch pollen [*n*, (%)]	699 (26.4)	582 (22.0)	1281 (48.4)
	Grass pollen [*n*, (%)]	803 (30.3)	774 (29.3)	1577 (59.6)
	House dust mite [*n*, (%)]	709 (26.8)	653 (24.7)	1362 (51.5)
	Cat [*n*, (%)]	384 (14.5)	325 (12.3)	709 (26.8)

*Note*: Patients tested with each allergen in skin test and/or specific IgE in absolute numbers [*n*] and percentage of the whole population (%) as well as arithmetic mean (x̄) and standard deviation (σ) of patient's age. This table includes also the tests of patients without an allergen‐specific history (compare Figure 1).

### 3.2 Comparing test sensitivity between extract‐based skin tests and molecular allergy testing by assessing specific IgE to four important allergens sources

**FIGURE 2:**
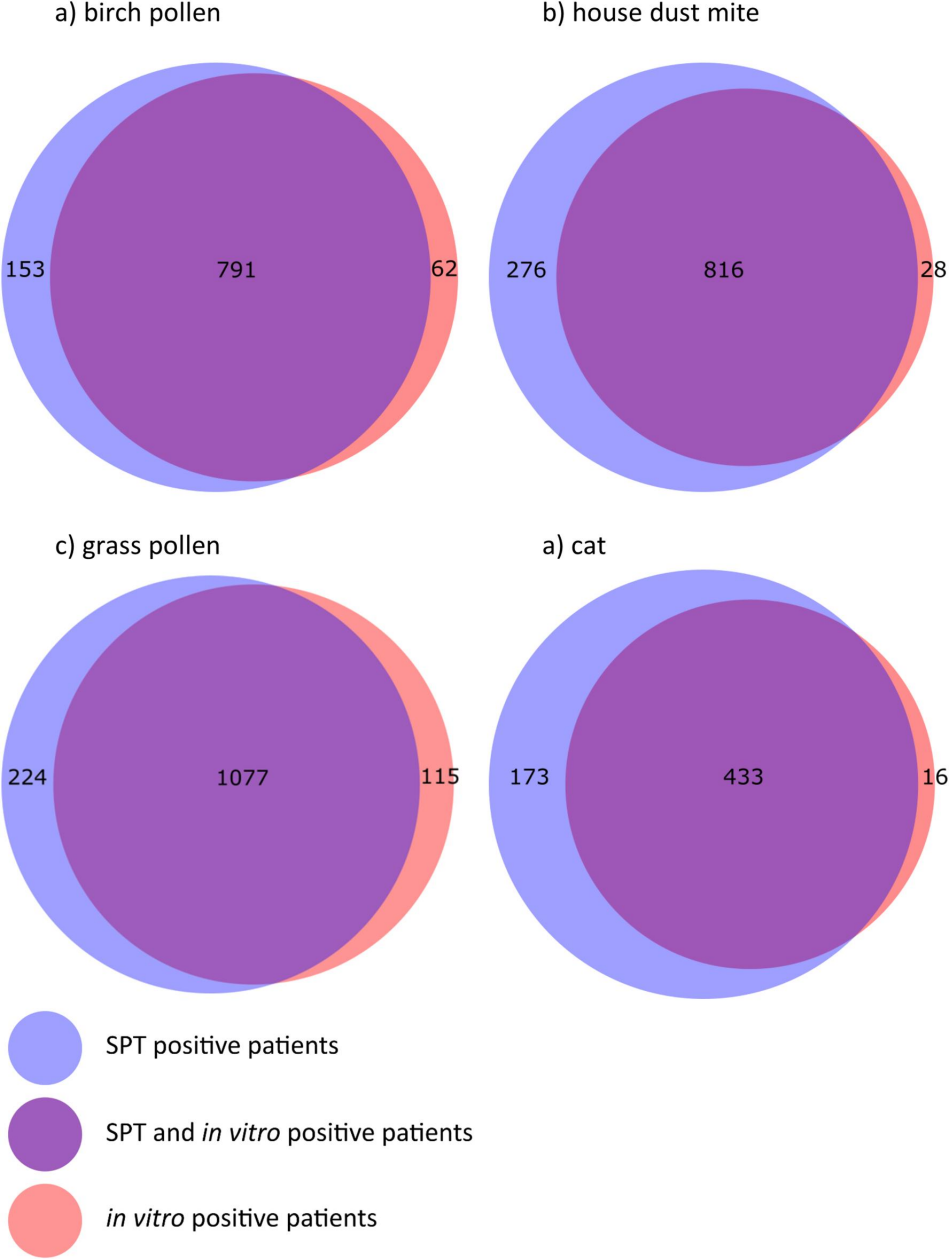
Venn diagrams of pairs of positive skin prick test or in vitro test in absolute numbers.

Only patients with at least one positive result in either SPT or sIgE were included for comparing the sensitivity of both methods. Concerning birch pollen sensitization, 791 (78.6%) out of 1006 patients with a clear‐cut history compatible with clinically relevant birch pollen allergy (symptoms from March to April) showed positive results with both methods, whereas 153 (15.2%) patients only had a positive result either with the SPT or 62 (6.2%) only with the sIgE to Bet v1 (*p* < 0.0001, Χ^2^ test, Figure 2A). Regarding history compatible with perennial house dust mite allergy, 816 (72.9%) out of 1120 patients had positive test results in SPT and sIgE, while 276 (24.6%) could only be detected by SPT versus 28 (2.5%) only by sIgE to the major allergens Der p1, Der p2 and/or Der p23 (*p* < 0.0001, Χ^2^ test, Figure 2B). Regarding grass pollen allergy with symptoms from May to July, 1077 (76.1%) out of 1416 sensitizations were discovered with both methods, 224 (15.8%) sensitizations were only detectable by SPT, while 115 (8.1%) only by sIgE to the two major allergens Phl p1/5 (*p* < 0.0001, Χ^2^ test, Figure 2C). Concerning perennial sensitization to the own pet cat or specific symptoms upon exposure to foreign cats, 433 (69.6%) out of 622 patients had positive results in both test systems, whereas the sensitization of 173 (27.8%) patients was only detectable through extract based SPT and only a minute number of 16 (2.6%) patients were discovered by sIgE to the major cat allergen Fel d1 (*p* < 0.0001, Χ^2^ test, Figure 2D).

### 3.3 Correlation between wheal size and antibody level

**FIGURE 3:**
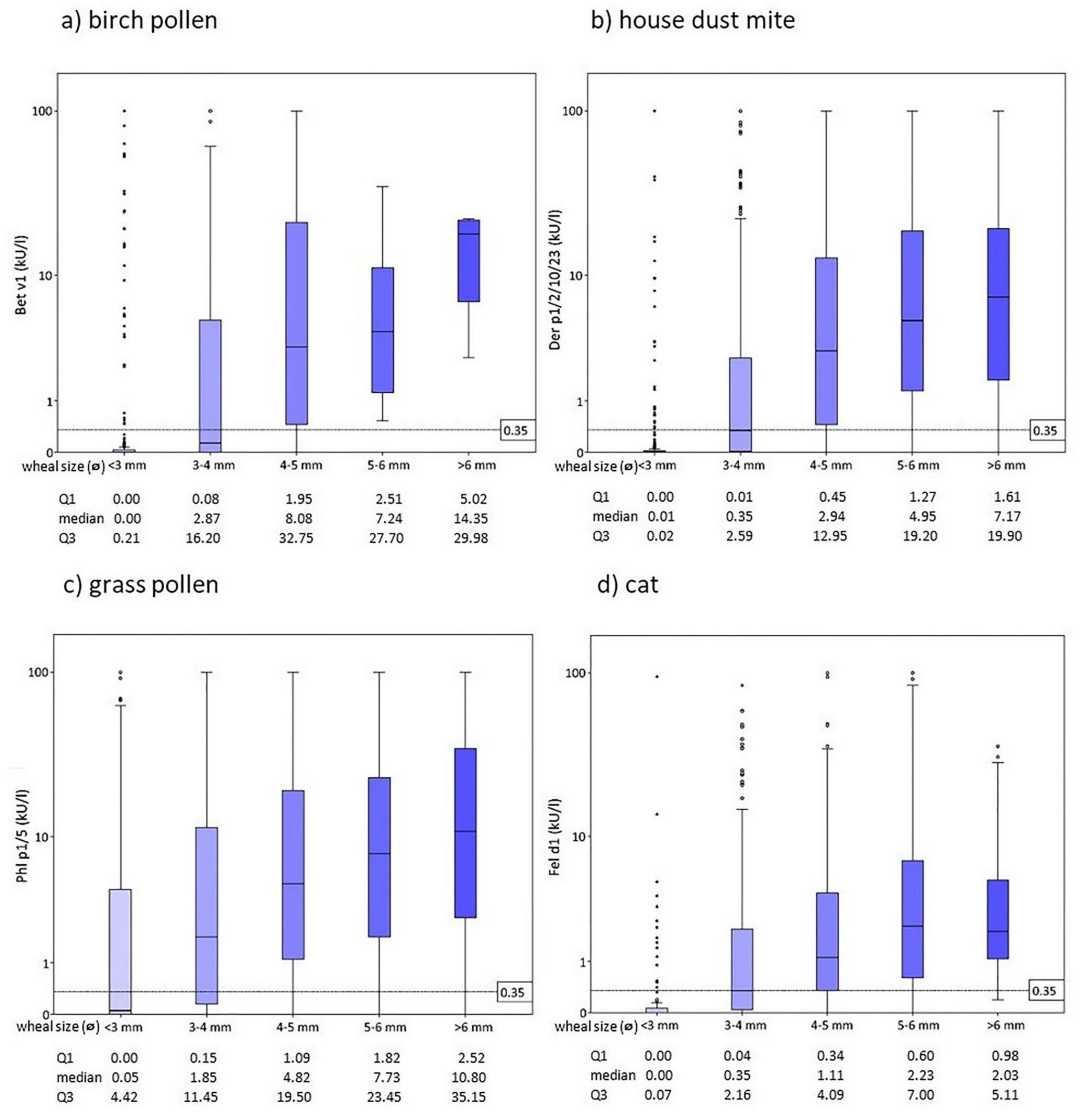
Box plots comparing antibody‐levels relative to the wheal size of the patients' skin prick test.

The correlation between wheal‐size categorized according to Ruëff et al.^7^ and sIgE levels was highest in dust‐mite, cat‐ and birch pollen sensitization (Spearman's Rho 0.54, 0.47 and 0.45, *p* < 0.01) and lowest in grass‐pollen sensitized individuals (0.35), while still retaining significance (*p* < 0.01). These data sets were also visualized with box plots, by which a positive correlation was visible for all four allergens (Figure 3).

### 3.4 The diagnostic value of adding Der p23 for diagnosing house dust mite allergy

**FIGURE 4:**
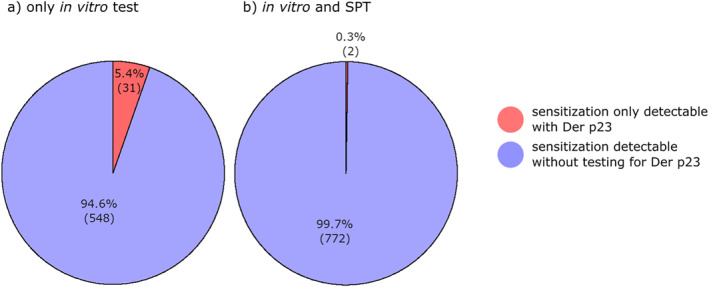
Pie chart demonstrating the low additional value of adding the major allergen Der p23 to the molecular allergen panel in house dust mite‐sensitized patients.

House dust mite extracts contain a lot of known allergens but only some are described as major allergens, namely Der p1, Der p2 and lately Der p23.^8^ We were interested in the additional value of adding Der p23 into the routine diagnostic panel. To begin with, dust mite‐sensitized patients were tested with Der p1/Der p2, including Der p23 and compared with Der p1/Der p2 without Der p23. Only 5.4% (31 of 579) showed positive Der p23 antibody titres with at the same time negative Der p1/2 results. Thus, the value of adding Der p23 into single allergen testing was relatively low. The other 94.6% (548) patients could have been detected by determination of Der p1 and Der p2 only. With the next step, we included SPTs into the analysis. Out of 776 detected sensitizations, only two patients (0.3%) were exclusively verifiable through Der p23 IgE antibodies, whereas 774 out of 776 (99.7%) would have been diagnosed by a combination of extract‐based SPTs and Der p1/2 only. This showed that the addition of Der p23 played only an insignificant role in increasing in vitro sensitivity in house dust mite allergy (Figure 4).

## 4 DISCUSSION

In this study we could demonstrate that the sensitivity of extract‐based SPT is superior to measurement of sIgE against the major allergens (also known as allergen “components”) of the most important inhalant allergen sources. This was true for complex allergen sources such as house dust mite with three (Der p1/2/23) and grass pollen with five (Phl p1/2/4/5/6) major allergens but also for less complex allergen sources with single dominating major allergens such as cat (Fel d1) or birch pollen (Bet v1).

Many allergologist regard in vitro testing with major allergens as the most modern approach of diagnosing type 1 allergy.^9^ However, allergen sources are often complex and consist of mixtures of major, minor and some pan‐allergens. If screening includes only the most prevalent variants of major allergens, allergic patients with sensitizations to minor allergens will produce false negative tests. Also, molecular allergens can be missing (unknown or commercially unavailable) for many rare allergens, for example, storage mites or shrimp.^10^, ^11^ This, after many years, keeps the door wide open for the standard allergy diagnostic pathway based on extracts from natural sources for skin tests but also in vitro tests. Extracts from natural sources offer the advantage of including a lot of minor allergen components and variants of the major allergens that are sometimes missing even in very well‐known allergens; for example, the major birch pollen allergen Bet v1 is currently listed in 28 isoforms on the IUIS Allfam Server.^12^ Also, most rare allergens are only available in extract form.^13^ Insufficient sensitivity of molecular allergology has always been an issue from the very beginning and has not been completely resolved for all allergens yet.^14^ However, extracts bear inherent disadvantages over molecular allergy diagnosis: (A) they come from natural sources and vary in their composition and quality^15^, ^16^ although in recent times, standardization has improved also the quality of commercial skin test extracts^17^; (B) they may contain cross‐reactive allergens producing clinically irrelevant results such as profilin or polcalcin; (C) cross‐reactive carbohydrate determinants (CCD) may produce positive in vitro tests to allergens derived from natural sources in extracts as well as highly purified molecular allergens.^18^

In the context of pollen‐associated food allergy, profilin from the date palm (Pho d2) and Lipid Transfer Protein from peach (Pru p3) have been available as purified commercial extracts for skin testing for more than a decade.^19^, ^20^ In a recent study, Asero and colleagues could show, that if molecular allergens are used for skin and in vitro tests side‐by‐side, sensitivity and specificity in LTP allergy to peach (Pru p3) is nearly the same, independently from the method used.^21^ However, this is the rare exception where allergen components are available for skin testing.

### 4.1 Comparing allergen‐specific test results

#### 4.1.1 Dust mites

Bousquet et al.^22^ had compared SPTs with extract based in vitro diagnostics using the same cut offs as in the FAZ. They published that out of 3140 sensitized patients, 57.6% (1810) got positive results in both tests, while 13.3% (419) of the sensitizations were only found with the SPT and 29.0% (911) only through in vitro diagnostics. They had, however, used allergen extracts not only for the skin but also the in vitro tests which may explain the differences. In the study of Bousquet et al., 81.2% of patients with a positive result in the SPT also had a positive in vitro‐test result, whilst in our study only 72.6% of the skin test positive patients had a positive in vitro test based on molecular allergy testing. In another much smaller study from Italy, SPTs were compared with the ISAC112 platform from the same manufacturer as in our study using a different technical platform but the same molecular allergens.^23^ The patients differed from our study as the Italian cohort also contained a lot of food allergic and urticaria patients that had been excluded in our study focusing on inhalant allergy. Also, Der p23 was missing in their 2019 version of the ISAC. The data are reported in a very different way to our study so that the results can be hardly compared. Adding the double positive and the double negative patients of the Italian study with 75.8% resulted in a very comparable level of agreement with our study focusing on the double positive results only and showing 72.9% agreement between skin and blood tests.

#### 4.1.2 Cat

Concerning cat sensitization, Bousquet et al.^22^ found 43.0% (668) of all sensitized patients had positive results in both tests, whilst 36.7% (571) sensitizations had only been found through in vitro diagnostics and 20.3% (316) only through SPT. Again, their results differed from our center as we had not employed cat extract but the major cat allergen Fel d1 in this study. In another recently published study with a different patient cohort, we had shown that the dominant cat‐allergen was Fel d1 with 93%. The additional Fel d2/4/7 sensitization had usually been caused by cross‐reactivity to other pet mammals.^24^ Concerning cat positive SPTs, the 71.5% (433 out of 606) in our center were quite similar to the 67.9% (668 out of 984) patients in the ECRHS cohort^22^ but lower than the 81.6% sum of SPT+/in vitro+ and SPT−/in vitro− patients in the Italian study.^23^

#### 4.1.3 Grass pollen

The results of grass pollen‐sensitized patients were compared with data generated by Knight et al.^25^ Out of 99 sensitized patients, 84% (84) had positive test results employing both techniques, while 11 sensitizations were only diagnosed by in vitro and only four by SPT. In the study of Knight et al.^25^ 95% patients (84) with a positive SPT also had a positive in vitro test, whilst only 83% (1078/1302) with a positive SPT in our own study also had a positive in vitro test. Besides other factors these differences could be explained by two different in vitro test methods and different cut‐offs in the two studies. Whilst antibody‐levels of 0.35 kU/l or higher with the ThermoFisher ImmunoCAP^®^ defined a positive result at the FAZ, antibody levels of 0.05 kU/l in the Hycor Hytec^®^288 had been considered positive in the study of Knight et al.^25^ The aforementioned Italian ISAC/SPT study was not comparable for grass and birch pollen as it had included sensitizations to the panallergens profilin and polcalcin that hardly ever cause symptomatic inhalative allergy.^23^

#### 4.1.4 Limitations

The study design followed a standard clinical approach. Due to the large number of 2646 included patients it was not possible to perform provocation tests as external “golden standard.” Hence, this study can only describe the proportion of patients with an allergen‐source specific history that could be detected by each of the tests. As negative controls had not been included into this study, it was not possible to calculate specificity, negative or positive predictive values.

An allergen‐source specific history may not be such specific. In Austria there are overlapping pollen seasons from March‐April for birch (*Betula* sp.—cross reactive with other species from the beech tree family e.g., hazelnut, Alder, oak and beech) and the less important allergen source ash tree pollen (*Fraxinus* sp.—cross reactive with olive tree pollen).^26^ The same overlap exists for the long grass pollen season from May to July with the less frequent fungal allergen sources *Alternaria* and *Cladosporium* spores. In a previous study, we detected co‐sensitization to cat and dog in 25.9% of the patients.^24^ A pet‐derived history cannot be clearly attributed to a single causative animal in households were both species are present.

#### 4.1.5 The disappearance of commercial skin test solutions

Nearly 15 years ago, the European guideline on allergen products EMEA/CHMP/BWP/304831/2007^27^ was introduced in order to increase the quality of commercially available SPT solutions. However, many of these SPT solutions have since been withdrawn from the market by the manufacturers which was pointed out in an Editorial by Asero and Cecchi.^10^ This happened because of various reasons but one of the most important ones are the high administrative costs for manufacturers for being able to keep them on the market.^28^ A recent EAACI survey showed that around 90% of European allergologists regard the SPT as the most important diagnostic tool.^29^ Germany had been the first European country to implement the European guideline into national law but in recent years, many European countries have followed the German example putting even more pressure into this difficult matter. Hence, the EAACI created a task force aiming at keeping a close eye on not to lose the prerequisite for one of the allergologist's most important tools: commercially available SPT solutions to frequent and rare allergens of good quality and constant availability.^30^

## 5 CONCLUSION

This study reveals that screening for inhalant allergy with even the best characterized molecular allergens has insufficient sensitivity and cannot simply replace extract‐based testing. Therefore, a combination of both—extract‐based skin test supplemented by in vitro tests—is recommended for retaining sufficient sensitivity in routine clinical practice.

## AUTHOR CONTRIBUTION

Tobias Gureczny analyzed the data, wrote the draft version of the manuscript, and conceived the Figures. Tobias Gureczny, Benjamin Heindl, and Livia Klug retrieved the patients' data during their diploma thesis. Felix Wantke proofread the manuscript. Wolfgang Hemmer proofread the manuscript and supported the data analysis. Stefan Wöhrl conceived the study, revised the manuscript, and supervised Tobias Gureczny during his diploma thesis.

## CONFLICT OF INTEREST

SW declares having received lecture fees from ThermoFisher Scientific. The other authors have nothing to declare.

## Supporting information

Supporting Information S1
