# Supplementary material for: Allergy screening with extract‐based skin prick tests demonstrates higher sensitivity over in vitro molecular allergy testing
Source: Clin Transl Allergy. 2023 Feb 5;13(2):e12220. doi: 10.1002/clt2.12220 (PMC9899493; doi:10.1002/clt2.12220)
Supplement: Supplementary file 1 — Supporting Information S1 [file CLT2-13-e12220-s001.doc]

## Einwilligungserklärung zur

## Verwendung alter Blutproben und vorhandener klinischer Daten

## für wissenschaftliche Studien

Name der Patientin/des Patienten: ............................................................................

Geburtsdatum: ...........................................

- Ich gebe hiermit mein freiwilliges Einverständnis, dass Reste vorhandener Blutproben, die mir im Rahmen meines Routine-Allergietests im Floridsdorfer Allergiezentrum (FAZ) abgenommen wurden, für wissenschaftliche Untersuchungen über Allergien weiterverwendet werden dürfen.

Für Laboruntersuchungen, die im Zuge von wissenschaftlichen Zusammenarbeiten außerhalb des FAZ durchgeführt werden, werden die Blutproben anonymisiert.

- Darüber hinaus willige ich ein, dass vorhandene klinische Informationen zu meiner Allergie sowie die im Rahmen der durchgeführten Routineallergietests erhobenen Befunde (z.B. Hauttestergebnisse, spezifisches IgE, Gesamt-IgE) für wissenschaftliche Auswertungen verwendet werden dürfen. Nur die Ärzte des FAZ und deren Mitarbeiter haben Zugang zu den vertraulichen Daten, in denen ich namentlich genannt werde. Diese Personen unterliegen der ärztlichen Schweigepflicht.

- Die Weitergabe der Daten erfolgt ausschließlich zu statistischen Zwecken und ich werde ausnahmslos darin nicht namentlich genannt. Auch in etwaigen Veröffentlichungen der Daten dieser klinischen Studien werde ich nicht namentlich genannt.

- Eine Kopie dieser Einwilligungserklärung habe ich erhalten. Das Original verbleibt beim Studienarzt.

......................................................................................................

(Datum und Unterschrift des Patienten bzw. des Erziehungsberechtigten)
